# A DNA aptamer with high affinity and specificity for molecular recognition and targeting therapy of gastric cancer

**DOI:** 10.1186/1471-2407-14-699

**Published:** 2014-09-23

**Authors:** Hong-Yong Cao, Ai-Hua Yuan, Wei Chen, Xue-Song Shi, Yi Miao

**Affiliations:** Department of General Surgery, Nanjing Hospital Affiliated to Nanjing Medical University, Nanjing, P. R. China; Department of General Surgery, First Affiliated Hospital of Nanjing Medical University, Nanjing, P. R. China

**Keywords:** Gastric cancer, DNA aptamer, Molecular probe, In vivo imaging, Live cell-SELEX

## Abstract

**Background:**

Aptamers have emerged as excellent molecular probes for cancer diagnosis and therapy. The aim of the current study was to determine the feasibility of using DNA aptamer cy-apt 20 developed by live cell-SELEX for detecting and targeting gastric cancer.

**Methods:**

The specificity, sensitivity and biostability of cy-apt 20 in detecting gastric cancer were assessed by binding assay, cell fluorescence imaging, and in vivo tumor imaging in animal model in comparison with non-gastric cancers.

**Results:**

Flow cytometric analysis showed that cy-apt 20 had higher than 78% of maximal binding rate to gastric cancer cells, much higher than that of non-gastric cancer cells. Cell fluorescence imaging and in vivo tumor imaging showed that the targeting recognition could be visualized by using minimal dose of fluorochrome labeled cy-apt 20. Meanwhile, strong fluorescence signals were detected and lasted for a period of time longer than 50 min in vitro and 240 min in vivo. The fluorescence intensities of gastric cancer were about seven folds in vitro and five folds of that of non-gastric cancers in vivo.

**Conclusion:**

Our study demonstrated that cy-apt 20 was an excellent molecular probe with high specificity and sensitivity and a certain degree of biostability for molecular recognition and targeting therapy of gastric cancer.

## Background

Disease biomarkers are widely used in medicine, but very few biomarkers are available for the diagnosis and targeting therapy of gastric cancer so far [1, 2]. Gastric cancer is a highly aggressive malignancy often diagnosed at an advanced stage [3]. Despite the decline in incidence and the major improvements in diagnosis and treatment, it remains the fourth commonest malignancy and the second leading cause of cancer death worldwide [3–5]. The carcinogenesis and progression of gastric cancer are determined by multi factors including Helicobacter pylori infection, activation of oncogenic pathways and epigenetic elements [6–8]. Genes and molecules participating in the proliferation, invasion, and metastasis of gastric cancer, such as growth factors and their receptors, cell-cycle regulators, cell-adhesion molecules and matrix-degrading enzymes, etc. are all considered as important determiners of prognosis [6–9]. It is desirable to identify useful biomarkers from these factors for diagnosing, stratifying, targeting gastric cancer and, ultimately, improve the survival of patients.

In the past two decades, great effort was made in search of reliable biomarkers to revolutionize the diagnosis and treatment of gastric cancer. By employing genomic, proteomic and metabolomic approaches, almost all genes and molecules participating in cancer growth, invasion and metastasis have been investigated as potential gastric cancer biomarker. However, few of these initially promising biomarkers have been validated for clinical use [1, 2, 7, 8, 10, 11]. The main challenge in identifying reliable biomarkers is the individual genetic variation and tumor heterogeneity, many aspects of which remain unknown yet [6–8, 11]. Other challenges include: the gene expression and protein products depend much on the cross talk of cancer cells, the genomic, proteomic and metabolomic approaches are often too complex and expensive to be applied in clinic at present time and biomarkers generated by such strategies are out of context of cancer cells [11–13].

**Figure 1:**
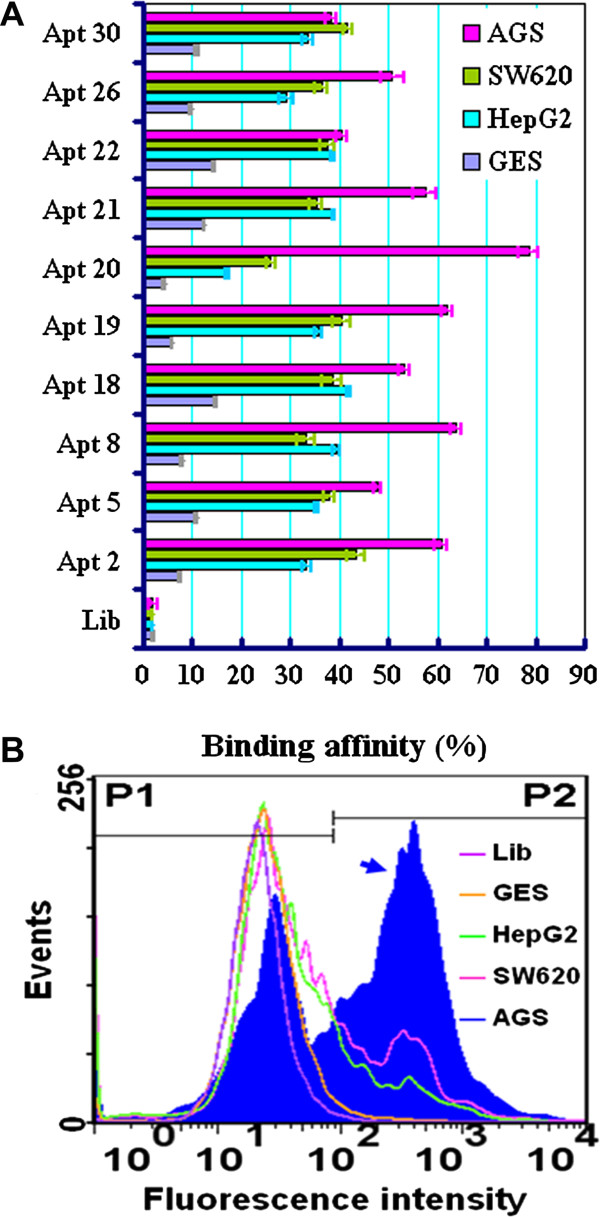
**Evolution of gastric carcinoma cell-specific aptamer cy-apt 20 by live cell-SELEX.** Human gastric carcinoma AGS cells were used as target cell for positive selections and human normal gastric epithelial GES-1 cells as negative cell for counter selections. The selection procedure was monitored by electrophoresis and flow cytometry analyses. Selected DNA aptamers were labled with FITC and their binding affinity to AGS cells were analyzed using flow cytometry. Lib to AGS cells and each identified ssDNA sequence to GES-1, HepG2 and SW620 cells were used as controls. The concentration of these FITC-labeled ssDNA used was 400 nM, and results were presented as mean ± standard error. **(A)** Ten ssDNA sequences with high binding ability to AGS were identified from the final pool. **(B)** Four of these ssDNA sequences were found have binding rates more than 60% to AGS cells, but only aptamer cy-apt 20 (arrow notified) have less than 30% binding affinity to non-gastric carcinoma HepG2 and SW620 cells. FITC: fluorescein isothiocyanate. Lib: FITC-labeled unselected library ssDNA. Apt or cy-apt: named identified ssDNA sequence. HepG2: human hepatocellular carcinoma cell. SW620: human colon carcinoma cell.

Recently, a new class of molecules termed aptamer has emerged as excellent molecular probes for cancer diagnosis and targeting therapy [14, 15]. Aptamers are single-stranded DNA (ssDNA) or RNA typically generated by an iterative screening process termed Systemic Evolution of Ligands by Exponential Enrichment (SELEX) [16]. The SELEX procedure involves progressive purification from a combinatorial library of nucleic acid ligands with a high affinity for a particular target by repeated rounds of partitioning and amplification [17]. In comparison with other molecular recognition elements, aptamers have the ability to bind specifically to a wide variety of targets ranging from small organic molecules to proteins [14, 15]. The basis for target recognition is the tertiary structures formed by the single-stranded oligonucleotides [18]. In addition, aptamers possess numerous advantageous characteristics, including small size, lack of immunogenicity, easy and reproducible synthesis, high binding affinity and molecular specificity, fast tissue penetration and low toxicity, tenability in binding affinity, and long-term stability [14, 15]. To generate cancer specific aptamers in context of cancer cells, an approach termed whole live cell based SELEX (live cell-SELEX) has been developed [19]. Accumulating evidences demonstrated that the live cell-SELEX is simple, fast, straightforward, reproducible, and most importantly, effective even when there is only a minor difference between a cancerous cell and an untransformed cell of the same tissue type [14, 15, 20, 21]. Thus far, a group of cancer specific aptamers were generated by using live cell-SELEX, some of them have been successfully used for cancer detection and targeting therapy [20–31].We have developed a gastric cancer specific DNA aptamer cy-apt 20 by employing live cell-SELEX. A series of experiments confirmed that, aptamer cy-apt 20 had higher than 70% of binding rate to gastric cancer cells and less than 30% of binding affinity to non-gastric cancer cells (unpublished data, see Figure 1A and B). The results indicated that the aptamer cy-apt 20 has great potential to be used for the management of gastric cancer. The aim of the current study was to determine the feasibility of using cy-apt 20 as a molecular probe for detecting and targeting gastric cancer.

## Methods

### Cell lines and cell culture

Human normal gastric epithelial cell line GES-1, gastric carcinoma cell line AGS, liver hepatoma cell line HepG2 and colon carcinoma cell line SW620 were obtained from American Type Culture Collection (ATCC; Manassas, VA). GES-1, AGS and SW620 cells were maintained and propagated in Dulbecco’s minimal essential medium (DMEM; Hyclone, Logan, UT) supplemented with 20% fetal bovine serum (FBS; Hyclone, Logan, UT) and 100 U/mL penicillin-streptomycin (Sigma, St. Louis, MO). HepG2 cells were maintained and propagated in RPMI-1640 (Sigma) supplemented with 10% FBS and 100 U/mL penicillin-streptomycin. All cells were cultured in 100 mm × 20 mm culture dishes at 37°C in a humidified atmosphere containing 5% CO_2_. All experiments were done using the >90% confluent cultures.

### DNA primers and libraries

Random DNA primers and library were designed using the Integrated DNA Technologies software (IDT, Coralville, IA), synthesized by standard phosphoramidite chemistry with an automated DNA synthesizer (3400 DNA Synthesizer; Applied Biosystems Inc, Foster City, CA) and purified by reverse phase High Performance Liquid Chromatography (RP-HPLC; Shanghai Sangon Biological Company, Shanghai, China). The purified library contained a central randomized sequence of 52 nucleotides (nt) flanked by two 18-nt primer hybridization sites (ATACCAGCTTATTCAATT-52-nt-AGATAGTAAGTGCAATCT). The forward and reverse primers used in the PCR performed in the process of cell-SELEX were separately labeled with fluorescein isothiocyanate (FITC) (5′-FITC-ATACCAGCTTATTCAATT-3′) and biotin (Bio) (5′-Bio-AGATAGTAAGTGCAATCT-3′) at the 5′ end in order to synthesize double-labeled and double-stranded DNA molecules.

### Procedure of cell-SELEX

AGS cells were used as target cell for positive selections and GES-1 as negative cell for the counter selections. The live cell-SELEX was performed and monitored according to the protocol reported elsewhere [19]. Briefly, before the selection, culture cells were washed twice with washing buffer (4.5 g/L glucose and 5 mM MgCl_2_ in Dulbecco’s phosphate buffered saline with calcium chloride and magnesium chloride) (Sigma). 200 pmol of library or DNA pool was dissolved in 400 μl of binding buffer (500 nM/L). The binding buffer was prepared by adding 0.1 mg/ml tRNA (Sigma) and 1 mg/ml of bovine serum albumin (BSA; Sigma) into washing buffer. The library or DNA pool was denatured and incubated with 5 × 10^6^ AGS cells at 4°C on rocker for 40 min. After incubation, the cells were washed three times with washing buffer to remove unbound sequences. The cell-DNA complex was resuspended in 400 μl binding buffer and heated at 95°C for 15 min and centrifuged at 14000 rpm to elute the bound DNAs. The eluted DNAs were then incubated with 1 × 10^7^ GES-1 cells for counter selection at 4°C on rocker for 40 min. The cells were then centrifuged at 14000 rpm for 5 min. The supernatant containing the ssDNA was recovered and amplified by polymerase chain reaction (PCR) using FITC- and biotin-labeled primers. Amplifications were carried out in an Eppendorf PCR thermocycler (Eppendorf GAC 22331, Hamburg, Germany). The selected sense ssDNA strands were separated from the biotinylated antisense ssDNA strands by alkaline denaturation and purified by streptavidin-coated sepharose beads (Amersham Biosciences). The selected ssDNA was then dried and resuspended in binding buffer for the next round of selection. After eight rounds of selections, the final selected ssDNA pool was PCR-amplified and cloned into Escherichia coli using the TOTO TA cloning kit (Invitrogen, Carlsbad, CA). Cloning of the PCR products and sequencing of the selected sense ssDNA were performed by Shanghai Sangon Biological Company (Shanghai, China).

### Flow cytometric analysis

To assess the enrichment of specific aptamer candidates and the binding capacity and affinity of the selected aptamer candidates to AGS cells, culture cells at 90% confluent were harvested by nonenzymatic cell dissociation solution (Sigma) and then washed twice with washing buffer. 5 × 10^5^ cells were incubated with varying concentrations of FITC-labeled selected ssDNA in 200 μL binding buffer on ice for varied time lengths. Cells were then washed twice with washing buffer and suspended in 200 μL washing buffer. The fluorescence was analyzed by flow cytometry (BD FACS Calibur, BD Biosciences). The FITC-labeled unselected ssDNA to AGS and the FITC-labeled selected ssDNA to GES, HepG2 and SW620 were used as controls. All the experiments were repeated 3 times. The mean fluorescence intensity of target cells labeled by selected ssDNA was calculated by subtracting the mean fluorescence intensity of produced by unselected ssDNA.

### Imaging of target cells with FITC-labled cy-apt 20

The specificity of the apatamer candidate cy-apt 20 in recognizing AGS cells was further visualized by fluorescence imaging. Both HepG2 and SW620 cells were used as controls. Before the imaging, culture cells in flat-bottomed 6-well plates (Costar, Corning, NY, USA) were washed twice with washing buffer and then incubated with 400 nM of FITC-labled cy-apt 20 in 200 μL binding buffer on ice for 40 min. After washing, the stained cells were viewed with an invert fluorescence microscope (TE2000, Nikon) using the standard-FITC filter set (excitation at 490 nm and emission at 520 nm). Pictures of the stained cells were taken with a DXM1200F digital camera (Nikon).

### BALB/c nude mice and xenograft model

Female BALB/c nude mice, 4–6 weeks old, were bred in the Experimental Animal Center of Nanjing Medical University. Institutional guidelines were followed in handling the animals. The animals were randomly assigned into three groups, each group contained three mice. The tumors were established by subcutaneous injection of 1 × 10^6^ AGS, SW620, and HepG2 cells in 200 μL PBS into the axillary region of the mice. After tumor imaging, the animals were euthanized, and tumor tissues were removed. Tumor tissues were then fixed in 10% buffered formalin, embedded in paraffin, and stained with hematoxylin and eosin for histopathological examination. The protocol was approved by the committee on the use of live animals in teaching and research at Nanjing Medical University.

### In vivo imaging of tumors with aptamer cy-apt 20

Mice xenograft model was established as described above. 200 μl of physiological saline containing different concentrations of cy-apt 20 labeled with Cy5 was injected via tail vein when the tumor grown visible for the assessment. Equivalent Cy5-labled Lib ssDNA was also used as controls. Fluorescence signals were imaged at different time points after injection of cy5-cy-apt 20 by IVIS Spectrum Imaging System (Caliper Life Sciences, Hopkinton, MA) with a 615–665 nm excitation filter and a 695–770 nm emission filter, respectively. The fluorescence signals of tumors relative to background were also measured and results were presented as fold changes vs. Background.

### Data processing and statistical analysis

The fluorescence was determined with a flow cytometry by counting 10,000 events per sample. Data were read and processed by FlowJo software (version 7.6 for Windows, Tree Star, Ashland, OR). Relative intensity of fluorescence signals of the imaged tumors were quantified using Image J software (version 1.47 for Windows, NIH, Bethesda, MD). Results were presented as mean ± standard error.

## Results

### Evolution of gastric carcinoma cell-specific aptamer cy-apt 20 by live cell-SELEX

The whole live cell SELEX strategy has been developed recently for generating aptamers against cancer cells. The live cell SELEX procedure is simple, fast, straightforward, reproducible, and can be done without prior knowledge of target molecules. We have adopted the live cell SELEX recently to generate aptamers against gastric cancer cells. In our study, human gastric carcinoma AGS cells were used as target cell for positive selections and human normal gastric epithelial GES-1 cells were used as negative cell for counter selections. The Live cell-SELEX procedure and monitoring processes were done as described elsewhere [19]. After twelve rounds of selection, the ssDNA sequences with better binding affinity to the target cells were being enriched (data not shown). By cloning, sequencing, and subsequent flow cytometric analyses, thirty potential ssDNA sequences named cy-apt 01–30 were identified as potential aptamer candidates specific to AGS cells (data not shown). Among them, ten sequences had high binding ability to AGS cells, four sequences had binding rates higher than 60% to AGS cells (Figure 1A), however, only cy-apt 20 (nucleotide sequence: CGACCCGGCACAAACCCAGAACCATATACAC GATCATTAGTCTCCTGGGCCG) had higher than 70% of binding rate to AGS cells and less than 30% of binding affinity to non-gastric cancer cells (Figure 1A and B).

### Characterization of aptamer cy-apt 20 in vitro

**Figure 2:**
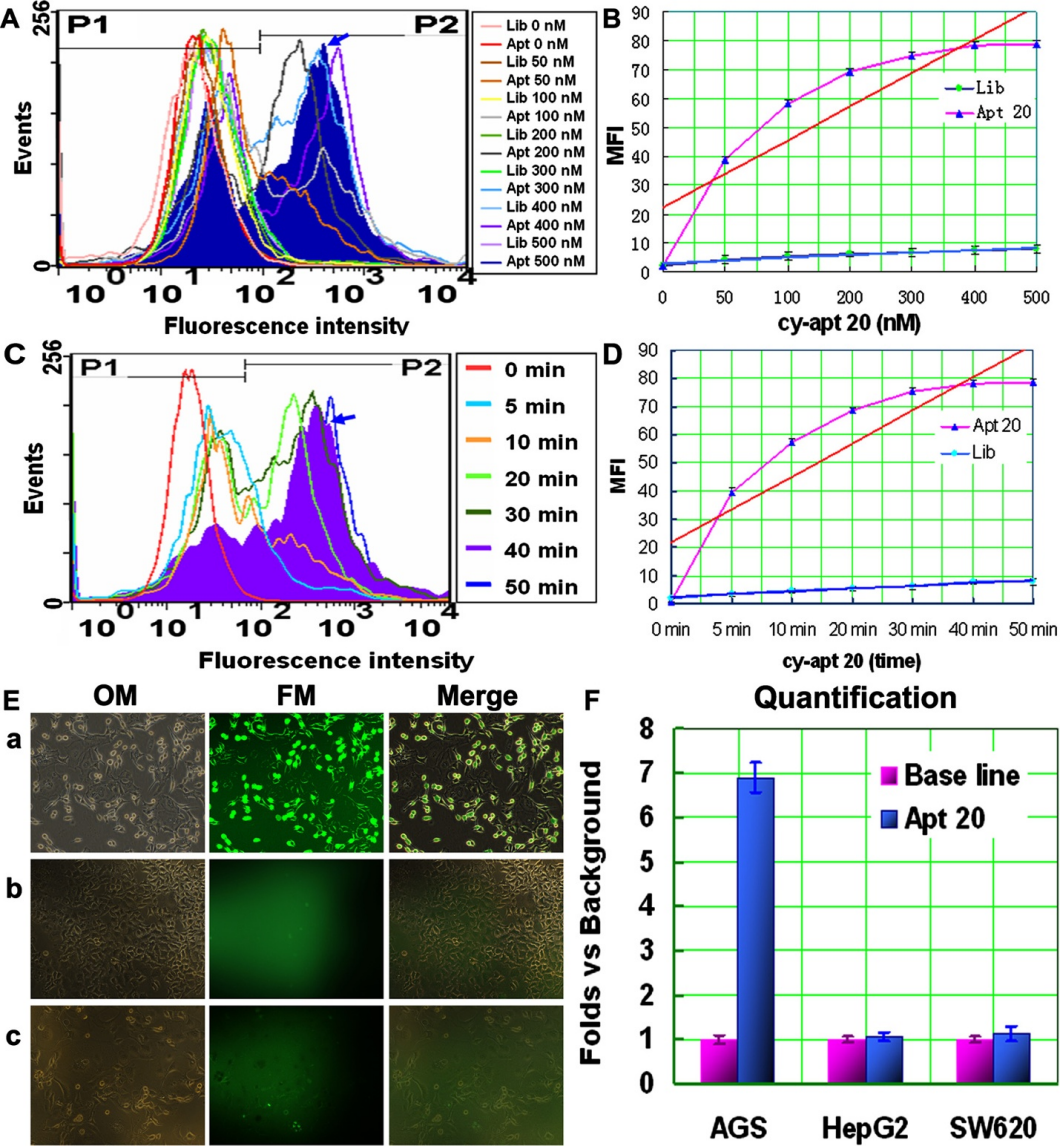
**Characterization of aptamer cy-apt 20 in vitro.** The specific binding capacity of aptamer cy-apt 20 to gastric carcinoma cells was also assessed with flow cytometry by varying the concentration of FITC-cy-apt 20 (from 0 nM to 500 nM) for varied time length of incubation (from 0 min to 50 min). Equivalent FITC-Lib ssDNA were used as controls and results were presented as mean ± standard error. **(A-B)** increased binding rates were seen with increasing the concentrations of FITC-cy-apt 20 after 40 min of incubation and peaked at 400 nM (arrow notified). **(C-D)** increased binding rates were also seen by increasing the incubation time length of AGS cells with 400 nM of FITC-cy-apt 20 (arrow notified) and peaked at 40 min. **(E)** The specificity of cy-apt 20 in recognizing AGS cells was further visualized by fluorescence imaging. All the three kinds of tumor cells were separately incubated with 400 nM of FITC-labled cy-apt 20 for 40 min, and then observed with an invert fluorescence microscope. (Ea) most of AGS cells were stained by FITC-labled cy-apt 20, whereas, few living HepG2 (Eb) and SW620 (Ec) cells exhibited detectable fluorescence. **(F)** The fluorescence signals of tumor cells relative to background were quantified using NIH Image J software and results were presented as fold changes vs. Background ± standard error. MFI: mean fluorescence intensity. OM: optical microscope; FM: fluorescence microscope.

The binding affinity and capacity of aptamer cy-apt 20 to AGS cells was assessed by flow cytometry. Equivalent library ssDNA were used as controls at the same conditions for cy-apt 20. The concentration of FITC-cy-apt 20 was first varied from 0 nM to 500 nM, and then the time length of incubation varied from 0 min to 50 min. The data showed that the fluorescence intensity of AGS cells was steadily increased after 40 min of incubation with increasing concentrations of FITC-cy-apt 20 (Figure 2A and B) and peaked at the concentration of 400 nM. The fluorescence intensity of AGS cells were also steadily increased with increasing incubation time length of AGS cells with FITC-cy-apt 20 and peaked at the time point of 40 min (Figure 2C and D). Meanwhile, the specificity of cy-apt 20 in recognizing AGS cells was further visualized by fluorescence imaging. The three kinds of tumor cells were separately incubated with 400 nM of FITC-labled cy-apt 20 for 40 min, and then observed with an invert fluorescence microscope. The result showed that most of AGS cells were stained by FITC-labled cy-apt 20 (Figure 2Ea), whereas, few living HepG2 (Figure 2Eb) and SW620 cells (Figure 2Ec) exhibited detectable fluorescence. The mean fluorescence intensity of AGS cells was approximately seven folds of that of HepG2 and SW620 cells (Figure 2F).

### Detection of gastric cancer in vivo using cy-apt 20

**Figure 3:**
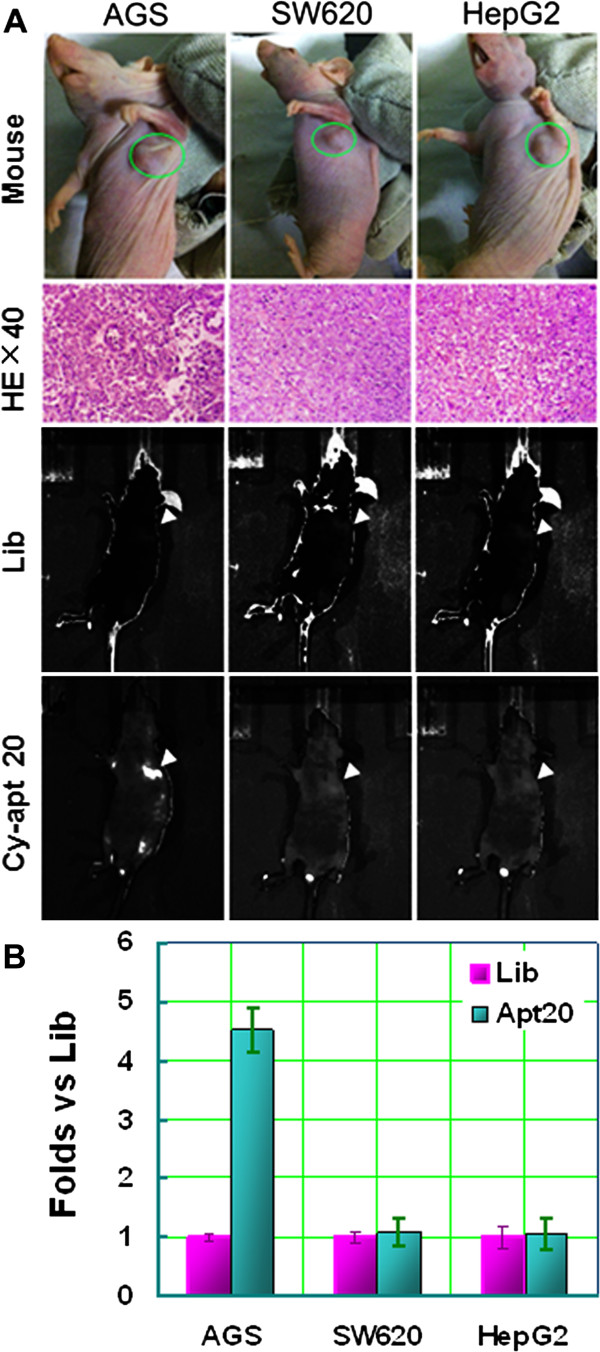
**Detection of gastric cancer in vivo using cy-apt 20.** The feasibility of using aptamer cy-apt 20 for detecting gastric cancer in vivo was observed by IVIS Spectrum Imaging System (Caliper Life Sciences, Hopkinton, MA). AGS cells were subcutaneously injected into the left axillary regions of BALB/c nude mice and HepG2 and SW620 were transplanted as controls. 200 μl of physiological saline containing 1 nM of cy-apt 20 labeled with Cy5 was injected via tail vein when the tumor grown visible for the assessment. Equivalent Cy5-labled Lib ssDNA was also used as control. **(A)** The top row show visible tumor formation in the left axillae of the mice (circle notified). The mid-upper row show histopathological examination of the tumor formation. Tumor tissues were embedded in paraffin and stained with hematoxylin and eosin for the examination. The mid-lower row show there was no fluorescence signal detectable in all tumor sites using Cy5-labled Lib ssDNA. The bottom row show there were strong fluorescence signals in AGS tumor site using Cy5-labled cy-apt 20 imaged at 60 min after injection (arrow notified), whereas there was no fluorescence signal detectable in either tumor site of HepG2 or SW620. **(B)** quantification of the signal-to-background ratios of the tumor imaged using Cy5-labled cy-apt 20. The fluorescence signals of tumor relative to background were measured using Image J software and results were presented as fold changes vs. Background ± standard error.

The feasibility of using aptamer cy-apt 20 for detecting gastric cancer in vivo was determined by mouse xenograft model with IVIS Spectrum Imaging System (Caliper Life Sciences, Hopkinton, MA). 200 μl of physiological saline containing 1 nM of cy-apt 20 labeled with Cy5 was injected via tail vein when the tumor grown visible for the assessment. Equivalent Cy5-labled Lib ssDNA was also used as control. Fluorescence signals of the tumors were imaged with IVIS Spectrum Imaging System at 60 min after injection (Figure 3A). The fluorescence signals of tumors relative to background were measured using Image J software (version 1.47 for Windows) and results were presented as fold changes vs. Background (Figure 3B). Histopathological examination was routinely performed to confirm the tumor formation when tumor imaging finished (Figure 3A). The results showed that there was no fluorescence signal detectable in all tumor sites imaged using Cy5-labled Lib ssDNA. Strong fluorescence signals were detected in AGS tumor site imaged using Cy5-labled cy-apt 20 (Figure 3AB, arrow notified), whereas, there was no fluorescence signal detectable in either tumor site of HepG2 or SW620.

### Characterization of aptamer cy-apt 20 in vivo

**Figure 4:**
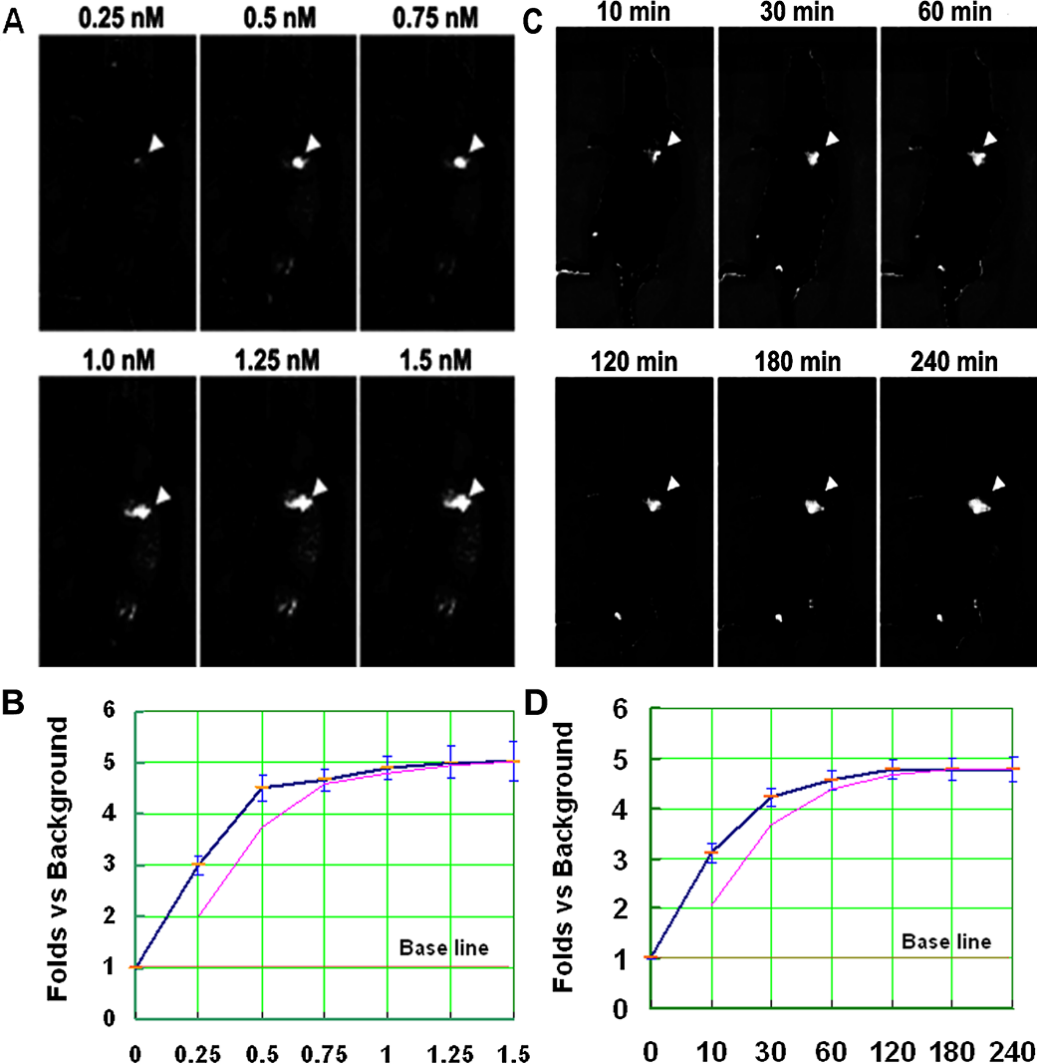
**Characterization of aptamer cy-apt 20 in vivo.** The efficacy of aptamer cy-apt 20 in detecting of gastric cancer in vivo was further determined by IVIS Spectrum Imaging System in dose and time changing manner. The fluorescence signals of tumors relative to background were measured using Image J software and results were presented as fold changes vs. Background ± standard error. **(A-B)** increased fluorescence signals were detected by increasing the concentration of cy5-labeled cy-apt 20 (from 0.25 nM to 1.5 nM) and peaked at 1.25 nM (arrow notified). **(C-D)** increased fluorescence signals were also detected by increasing imaging interval (from 10 min to 240 min) at the concentration of 1 nM of cy5-labeled cy-apt 20 and peaked at 120 min (arrow notified).

The efficacy of aptamer cy-apt 20 in detecting of gastric cancer in vivo was further determined by IVIS Spectrum Imaging System in a dose and time changing manner. The fluorescence signals of tumors were measured in the same manner as described above. Measurable fluorescence signals were detected at 0.25 nM, then increased with increasing concentration of cy5-labeled cy-apt 20 (from 0.25 nM to 1.5 nM), and peaked at the concentration of 1.25 nM (Figure 4AB, arrow notified). Meanwhile, fluorescence signals began to be detectable 10 min after administration of 1 nM cy5-labeled cy-apt 20, then steadily increased by increasing the imaging interval (from 10 min to 240 min), and peaked at 120 min (Figure 4CD, arrow notified).

## Discussion

The live cell-SELEX is proved to be a simple, but effective, reproducible, and widely applicable approach in generating high-affinity aptamers without prior knowledge of target molecules on tumor cells [19–22]. A large number of useful aptamers generated by this method are applied in the study of tumor biology, and even in diagnosis and targeting therapy of cancers [23–31]. The encouraging results obtained with aptamers combined with their intrinsic properties and the versatility of the live cell-SELEX procedure have inspired us to employing this method to develop gastric cancer specific DNA aptamers. In our study, human gastric carcinoma AGS cell was used as target cell for positive selections and human normal gastric epithelial GES-1 cell was used as negative cell for counter selections. Through twelve rounds of successive selections, a pool of ssDNA containing sequences with higher binding affinity to the target cells has been enriched. By cloning and sequencing, we identified an ssDNA sequence from the final ssDNA pool named cy-apt 20 as potential gastric cancer specific aptamer (Figure 1A). Compared experiments subsequently demonstrated that cy-apt 20 had higher than 70% of binding rate to AGS cells and less than 30% of binding affinity to non-gastric cancer cells (Figure 1B). The data (unpublished yet) indicated that cy-apt 20 may be a useful tool for detecting and targeting gastric cancer.

In the present study, we were to determine the feasibility of using cy-apt 20 as a molecular probe for detecting gastric cancer. The binding affinity and stability of cy-apt 20 in recognition of AGS cells were first assessed by binding assay in dose and time length changing manners, as a molecular tool for detecting target cancer cells must possess tenable binding affinity and stability in addition to high specificity [1, 2, 10, 12, 13]. Equivalent library ssDNA were used as controls at the same conditions of cy-apt 20. Flow cytometric analysis showed that cy-apt 20 had higher than 78% of maximal binding rate to gastric cancer cells, much higher than that of non-gastric cancer cells. The fluorescence intensity of AGS cells was steadily increased after 40 min of incubation with increasing concentrations of FITC-cy-apt 20 and peaked at the concentration of 400 nM (Figure 2A-B). So was the fluorescence intensity of AGS cells increased by increasing the time length of incubation with 400 nM of FITC-cy-apt 20, peaked at the time point of 40 min, and lasted for a period of time longer than 50 min (Figure 3C-D). The results demonstrated that targeting recognition can be established by using minimal dose of cy-apt 20 and lasted for a period of time long enough for detections.

To further ascertain the feasibility of using aptamer cy-apt 20 for detecting gastric cancer cells in vitro, fluorescence imaging was performed in comparison with non-gastric cancer cells. Because a best biomarker based diagnosis must be produced in direct, simplified and visualized ways [1, 2, 10, 12, 13]. In the compared examination, AGS cells were incubated with 400 nM of FITC-labled cy-apt 20 for 40 min, both human hepatocellular carcinoma cell HepG2 and colon carcinoma cell SW620 were used as controls. After incubation, the stained cells were viewed with an invert fluorescence microscope. The imaging showed that most of the AGS cells were stained by FITC-labled cy-apt 20 (Figure 2Ea), whereas, few HepG2 (Figure 2Eb) and SW620 (Figure 2Ec) cells were stained by FITC-labled cy-apt 20. The fluorescence intensity of AGS cells was approximately seven folds of that of HepG2 and SW620 cells (Figure 2F). The results further indicated that cy-apt 20 may be a useful molecule tool for detecting and targeting gastric cancer cells.Next, a mice xenograft model was established to determine the feasibility of using cy-apt 20 for detecting gastric cancer in vivo. AGS cells were subcutaneously injected into the left axillary regions of BALB/c nude mice and both HepG2 and SW620 were transplanted as controls. 200 μl of physiological saline containing 1 nM of cy-apt 20 labeled with Cy5 was injected via tail vein for in vivo imaging when the tumor grown visible for the assessment. Equivalent Cy5-labled Lib ssDNA was also used as control (Figure 3A). In vivo imaging showed that there was no fluorescence signal detectable in all tumor sites 60 min after the injection of Cy5-labled Lib ssDNA. Strong fluorescence signals were detected in AGS tumor site after the injection of Cy5-labled cy-apt 20, whereas, no fluorescence signal was detected in either site of HepG2 or SW620 tumors. The fluorescence signals of AGS tumors imaged using Cy5-labled cy-apt 20 was approximately five folds of that of controls (Figure 3B).The efficacy of using aptamer cy-apt 20 in detecting gastric cancer in vivo was also assessed in dose and time length changing manners. Strong fluorescence signals were detected after injection of 0.25 nM cy5-labeled cy-apt 20, increased by increasing the concentration of cy5-labeled cy-apt 20, and peaked at 1.25 nM (arrow notified) (Figure 4A-B). Strong fluorescence signals were also detected 10 min after injection of 1 nM cy5-labeled cy-apt 20, increased by increasing the imaging interval, and peaked at 120 min (arrow notified), and lasted for a period of time longer than 240 min (Figure 4C-D). The results further demonstrated that aptamer cy-apt 20 possessed excellent specificity and sensitivity to target cells with a certain degree of biostability in vivo.

## Conclusion

We demonstrated by a series of experiments that cy-apt 20 was an excellent molecular probe with a certain degree of biostability and high specificity and sensitivity for molecular recognition and targeting therapy of gastric cancer. Further studies will be necessary to determine the feasibility of using cy-apt 20 as a vehicle for drug delivery.
